# Development of a Luminex Bead Based Assay for Diagnosis of Toxocariasis Using Recombinant Antigens Tc-CTL-1 and Tc-TES-26

**DOI:** 10.1371/journal.pntd.0004168

**Published:** 2015-10-20

**Authors:** John P. Anderson, Lisa N. Rascoe, Keith Levert, Holly M. Chastain, Matthew S. Reed, Hilda N. Rivera, Isabel McAuliffe, Bin Zhan, Ryan E. Wiegand, Peter J. Hotez, Patricia P. Wilkins, Jan Pohl, Sukwan Handali

**Affiliations:** 1 Division of Parasitic Diseases and Malaria, Centers for Disease Control and Prevention, Atlanta, Georgia, United States of America; 2 National Center for Emerging and Zoonotic Infectious Diseases, Centers for Disease Control and Prevention, Atlanta, Georgia, United States of America; 3 National School of Tropical Medicine, Baylor College of Medicine, Houston, Texas, United States of America; Universidade Federal de Pelotas, BRAZIL

## Abstract

The clinical spectrum of human disease caused by the roundworms *Toxocara canis* and *Toxocara cati* ranges from visceral and ocular larva migrans to covert toxocariasis. The parasite is not typically recovered in affected tissues, so detection of parasite-specific antibodies is usually necessary for establishing a diagnosis. The most reliable immunodiagnostic methods use the *Toxocara* excretory-secretory antigens (TES-Ag) in ELISA formats to detect Toxocara-specific antibodies. To eliminate the need for native parasite materials, we identified and purified immunodiagnostic antigens using 2D gel electrophoresis followed by electrospray ionization mass spectrometry. Three predominant immunoreactive proteins were found in the TES; all three had been previously described in the literature: Tc-CTL-1, Tc-TES-26, and Tc-MUC-3. We generated *Escherichia coli* expressed recombinant proteins for evaluation in Luminex based immunoassays. We were unable to produce a functional assay with the Tc-MUC-3 recombinant protein. Tc-CTL-1 and Tc-TES-26 were successfully coupled and tested using defined serum batteries. The use of both proteins together generated better results than if the proteins were used individually. The sensitivity and specificity of the assay for detecting visceral larval migrans using Tc-CTL-1 plus Tc-TES-26 was 99% and 94%, respectively; the sensitivity for detecting ocular larval migrans was 64%. The combined performance of the new assay was superior to the currently available EIA and could potentially be employed to replace current assays that rely on native TES-Ag.

## Author Summary

The roundworms *Toxocara canis* and *Toxocara cati* cause a broad spectrum of clinical disease in humans. Children are at particular risk of toxocariasis when they play in areas potentially contaminated with *Toxocara* eggs, such as playgrounds or sandboxes and ingest embryonated roundworm eggs. Currently, diagnosis for toxocariasis relies on clinical signs, history of exposure to puppies or kittens, laboratory findings (including eosinophilia), and the detection of antibodies to *Toxocara* antigens. The enzyme immunoassay using *T*. *canis* excretory secretory antigens from infective-stage larvae is the most useful diagnostic test for toxocaral visceral larva migrans (VLM) and ocular larva migrans (OLM) and is the preferred assay used by most laboratories in the U.S. and worldwide. Although the EIA has been robust and reliable, improvement should be made in the specificity of the assay and the availability of a consistent antigen source. The crude TES-Ag shows cross-reactivity with antibodies from other common helminth infections of humans which reduces the usefulness of native, unfractionated TES Ag-based serodiagnosis in regions where poly-parasitism is endemic. To improve the assay performance, target antigenic proteins from *T*. *canis* excretory secretory antigens were identified using 2D gel electrophoresis. Three antigenic proteins sequences were found, expressed, and developed into Luminex bead-based assays. The combined use of two recombinant antigens (Tc-CTL-1 and Tc-TES-26) represents an improvement over the existing immunodiagnostic methods that rely on native parasite materials. In the future, additional antigens could be added from other parasites that cause larval migrans to form a single method for detecting larval migrans syndromes.

## Introduction

The roundworms *Toxocara canis* and *Toxocara cati* cause a broad spectrum of clinical disease in humans ranging from visceral and ocular larva migrans to covert and common toxocariasis. Children are at particular risk of toxocariasis when they play in areas, potentially contaminated with *Toxocara* eggs, such as playgrounds or sandboxes and ingest embryonated roundworm eggs. After ingestion, the eggs hatch in the gut and larvae disseminate hematogenously to the lungs, liver, muscle, brain, and/or eyes. Once in the tissues, the larvae are unable to continue their normal life cycle, and a local inflammatory response to larvae leads to the varied symptoms of toxocariasis (visceral, ocular larva migrans, and covert toxocariasis), and can lead to cerebritis and eosinophilic meningitis when larvae enter the central nervous system [1–6]. A third so-called “covert” form of toxocariasis has been linked to more subtle pulmonary and cognitive dysfunctions [7–9],and even educational deficits [10].

Currently, diagnosis for toxocariasis relies on clinical signs, history of exposure to puppies or kittens, laboratory findings (including eosinophilia), and the detection of antibodies to *Toxocara* antigens. The enzyme immunoassay (EIA) using *T*. *canis* excretory secretory antigens (TES-Ag) from infective-stage larvae is the most useful diagnostic test for toxocaral visceral larva migrans (VLM) and ocular larva migrans (OLM) and is the preferred assay used by most laboratories in the U.S. and worldwide [4, 11].The TES-Ag EIA has proven to be robust and reliable, although questions about specificity and reduced sensitivity leave ample room for improvement in laboratory diagnosis of toxocariasis [4, 6, 11]. In temperate countries, TES-Ag EIA and TES-western blot can provide sufficient support for clinical suspicion; however, testing is not widely available because of the limited availability of antigen made from *T*. *canis* larvae. The proven TES-Ag cross-reactivity with antibodies from other common helminth infections of humans also reduces the usefulness of native, unfractionated TES-Ag-based serodiagnosis in regions where poly-parasitism is endemic [6]. Recent efforts have focused on identification of recombinant proteins to improve sensitivity and specificity and to reduce reliance on native parasite materials [4]. Several groups have cloned, expressed, and developed EIAs based on recombinant antigens of the assay [12–16].Ultimately, a diagnostic method that utilizes one or more recombinant diagnostic antigens could result in improved assays that are more widely accessible to health care providers.

Our study aim was to identify one or more immunoreactive proteins found in the TES product from *T*. *canis* infective larvae and to develop at least one of these recombinant proteins as a diagnostic reagent in a multiplex bead format assay (Luminex) that could replace TES-Ag.

## Materials and Methods

### Ethics statement

All clinical samples used in this study were collected in previous studies with specific permission for future use of stored samples (CDC Study Protocol Number 3580). Samples were anonymized and the study was performed in compliance with protocols approved by the ethical review boards of the CDC.

### Serum specimens

Four sets of defined sera were used: (1) Two hundred and four sera from cases with presumed *Toxocara spp*. visceral larval migrans (VLM), based on the presence of clinical symptoms and signs and reactivity in the in-house TES-Ag EIA [11] and TES-Ag Western blot;(2) Fifty sera from cases of presumed *Toxocara spp*. ocular larval migrans (OLM) based on the presence of clinical symptoms and signs only (positivity with TES-Ag Western blot is not required to define OLM); (3) A control group consisting of two hundred and eighty eight sera from healthy U.S. residents; (4) A convenience panel for cross-reactivity evaluation consisting of one hundred and twenty U.S. patients infected with various infections other than *Toxocara spp*. and fourteen sera from Egyptians negative to *Toxocara spp*. Each sample was not exhaustively tested for all other parasites. Sets number 3 and number 4 were tested with TES-Ag Western blot.

Five serum samples collected from people with no travel history outside the United States were pooled and used as the ‘normal’ control serum pool. For TES 2D gel, we used a positive anti-*Baylisascaris procyonis* serum from a baboon that developed severe neural larval migrans after experimental infection with embryonated *B*. *procyonis* eggs. A *Toxocara*-positive serum pool was prepared by combining in equal volume of 10 EIA—positive serum samples.

### TES 2D gel electrophoresis and mass spectrometry (MS)

For 2D gel electrophoresis and mass spectrometry analysis we utilized TES-Ag provided by Dr. Steven Kayes’ Laboratory at the University of South Alabama, Mobile, AL. Briefly, larvae from artificially de-shelled embryonated *T*. *canis* eggs were cultured in RPMI 1640 at 37°C with saturated humidity and gassed with 5% CO_2_. The culture supernatants containing TES-Ag were collected, pooled, and concentrated [17]. Protein concentrations were determined using the Bradford Protein Assay and BCA Protein Quantification Assay (Pierce Biotechnology Rockford, IL). The 2D gel electrophoresis was performed following a protocol used previously [18]. Fifty μg of TES-Ag sample was separated on 11 cm, non-linear, pH 3–10 gradient Immobilized pH Gradient (IPG) strips (Bio-Rad, Hercules, CA) and after isoelectric focusing, the second dimension was carried out using Criterion XT 4–12% Bis-Tris pre-cast sodium dodecyl sulfate-polyacrylamide gel electrophoresis (SDS-PAGE) gels. Three of the 2D gels were transferred to nitrocellulose membranes and blotted and probed with a 1:50 dilution of a strong EIA positive *Toxocara* human sera pool, negative human serum sample, or a *B*. *procyonis* positive serum; the fourth gel was stained using a mass-spectrometry compatible silver stain. Proteins were chosen based on the comparative reactivity seen in the blots and were manually excised from silver-stained 2D gels. The target proteins were digested with trypsin and the resulting peptides analyzed by electrospray ionization mass spectrometry [18].The Mascot program was used to identify proteins from peptide sequence databases from the mass spectrometry data.

### Recombinant Antigen Preparation

#### Tc-CTL-1

The Tc-CTL-1 antigen is a 32 kDa C-type lectin secreted by *T*. *canis* larvae [19, 20]. The DNA encoding for Tc-CTL-1, minus the signal peptide at N-terminus was amplified from *T*. *canis* larval cDNA library [21], subsequently subcloned in-frame into the *E*. *coli* expression vector pET41a (Novagen) with fusion *Schistosoma japonicum* Glutathione S-transferase (GST) deleted and 6 His-tag expressed at C-terminus (NdeI/XhoI). The correct open reading frame (ORF) was confirmed by double stranded sequencing using the vector flanking primers (T7 promoter/T7 terminator). For expression of recombinant Tc-CTL-1 in bacteria, the recombinant pET41a was transformed into BL21(DE3) *Escherichia coli* (Novagen) and recombinant protein was expressed as insoluble inclusion bodies after being induced with 1 mM Isopropyl β-D-1-thiogalactopyranoside **(**IPTG) at 30°C overnight. The induced cells were collected by centrifugation at 5,000 g for 10 min at 4°C and lyzed with PBS, pH 7.4 containing 1 mg/mL lysozyme, 30 μg/mL DNAse I and 1% Triton-X 100, then sonicated for 60 seconds on 75% power for 3–5 times with 2 minutes interval. The inclusion bodies were washed with PBS, pH 7.4 containing 0.5% Triton X-100 for three times and then were solubilized in PBS, pH 7.4 + 8M urea and the recombinant protein was purified with immobilized metal affinity chromatography (IMAC) as described [22]. The recombinant Tc-CTL-1 was stored in 1 x PBS, pH7.4 +6M urea.

#### Tc-TES-26

The cloning of Tc-TES-26 was reported previously [23].We optimized and synthesized TES-26 gene based on UniProtKB/Swiss-Prot: P54190.1sequence for bacterial expression system [23] through a commercial company (Genscript). The plasmid, pGS21a-TES26, was transformed into BL21(DE3) *E*. *coli* under selection of 35 μg/mL chloramphenicol and 100 μg/mL ampicillin. The cultures were incubated in 30°C at 225 rpm. Once cell density reached to optical density (OD) = 0.6 at 600 nm, 1 mM IPTG was introduced for induction at 37°C for 3 hours. After IPTG induction, the bacterial culture was harvested by centrifugation at 5,000 rpm, 4°C for 20 minutes. The pellet was resuspended in PBS + 1% Triton X-100 + lysozyme 1 mg/mL. The mixture was incubated on ice for one hour, and then sonicated for 2 minutes at 65% power using a Misonix S-4000 ultrasonic liquid processor. After centrifugation, the supernatant contained the soluble target protein. The recombinant GST fusion protein was purified using glutathione Sepharose 4B affinity column (GE Healthcare) and the GST tag from the fusion protein was not deleted for future coupling uses. Protein concentration was determined using Bradford Protein Assay.

#### Tc-MUC-3

Tc-MUC-3 belongs to the TES-120 family of glycoproteins secreted by *T*. *canis*. The protein contains a repetitive serine/threonine-rich tract, and 4 of 36-amino acid six-cysteine (SXC) domains [24],and the sequence (AAD49340.1) was reported by Tetteh et al. [21].The gene was cloned into the pGs21a expression vector and the protein was expressed by a commercial company (Genscript, Piscataway, NJ). The plasmid was transformed into BL21(DE3) *E*. *coli* and grew under selection of 100 μg/mL ampicillin at 37°C with shaking at 200 rpm. Once the cell density reached to OD = 1.3 at 600 nm, the induction was done with 1 mM IPTG at 15°C overnight with shaking at 200 rpm. Cells were collected by centrifugation at 8,000 g, 4°C for 20 minutes, and the wet pellets were resuspended with 120 mL of PBS, pH 7.4 + 1 mM dithiothreitol (DTT), 1 mM PMSF. After sonication at 500 W for 3 seconds, on ice 6 seconds for a total of 30 min, cell pellets were spun down at 13,000 rpm, 4°C for 30 min. After centrifugation, the pellet was resuspended with 30 mL PBS, pH 7.4 + 8 M urea. After sonication at 500 W for 3 seconds, on ice 6 seconds for a total of 10 minutes, cell pellets were spun down at 13,000 rpm, 4°C for 30 min. After centrifugation, the solubilized pellet was loaded onto 3 mL pre-equilibrated nickel affinity column. The protein was washed with 30 mL PBS, pH 7.4 + 20 mM imidazole + 8 M urea. The protein was eluted with 30 mL PBS, pH 8.0 + 50 mM imidazole + 8 M urea and then with 30 mL PBS, pH 8.0 + 500 mM imidazole, 8 M urea. The eluted proteins were then loaded onto 3 mL pre-equilibrated Q Sepharose Fast Flow column (GE Life Sciences, Catalog No. 17-0510-10). The flow through was pooled and refolded against PBS, pH 7.4 + 4 mM DTT at 4°C. After dialysis, the sample was centrifuged at 13,000 rpm for 30 min and filtered through 0.22 μm membrane. The GST and HIS tags were left intact from the fusion protein. The protein concentration was determined using the Bradford Protein Assay.

### Luminex assay development

#### Protein coupling to MagPlex magnetic beads

All coupling procedures were carried out at room temperature (22°C). Recombinant proteins were coupled to MagPlex Magnetic Microspheres (Luminex, Austin; product # MC10026-01 and #MC10066-1) using 1-ethyl-3-[3-dimethylaminopropyl] carbodiimide hydrochloride (EDC)- *N-*hydroxysulfosuccinimide (Sulfo NHS) reactions [25, 26]. Briefly, beads were washed and activated by using 50mM 2-(*N-*morpholino)-ethanesulfonic acid (MES), pH 5, buffered saline (+ 0.85% NaCl and 0.05% Tween-20). After 40 minutes of incubation using end-over-end mixing at dark with Sulfo-NHS and EDC, beads were washed 2 times with MES buffered saline. The activated beads were then transferred to a new tube and washed once more. Beads were resuspended in the MES buffer without Tween-20 and 1.25 μg of each protein/1.25 x 10^6^beads were added. The total volume of reaction was brought to 500 μL with MES buffer without Tween-20. The coupling was performed for 3 hours in the dark at room temperature by end-over-end mixing. Beads were blocked with blocking buffer (PBS + 1% BSA + 0.05% sodium azide (NaN_3_), pH 7.4) for 30 minutes. The coupled beads were stored at 4°C in PBS + 1% BSA + 0.05% NaN_3_ + 0.05% Tween-20 + phenylmethylsulfonyl fluoride (PMSF) (1:500), pepstatin (1:1,000), leupeptin (1:1,000). The concentration of the beads was determined by using hemacytometer.

#### MagPlex immunoassay

All assays were carried out at room temperature (22°C). Fifty μL of the working microsphere mixture (50 beads/μL in PBS + 0.3% Tween-20 + 5% non-fat dry skim milk) and 50 μL of diluted sera (1:100 dilution in PBS + 0.3% Tween-20 + 5% non-fat dry skim milk) were added to 96-well black, round-bottom plates (Costar, Catalog No. 3792, Fisher Scientific). After 30 minutes incubation at room temperature with shaking (~800 rpm), the beads were washed using Biotek Magnetic Washer ELx50 (2 minutes magnetic separation, and then 2 cycles of dispensing 100 μL of PBS + Tween-20 0.3%, soaks for 40 seconds before aspiration). Bound antibodies were detected using 50 μL of biotinylated mouse anti-human IgG (clone H2, affinity purified, Southern Biotech, Birmingham, AL, Catalog No. 9042–08) diluted 1:200 in PBS + 1% BSA +0.05% NaN_3._ After 30 minutes incubation, the beads were washed as before. As a detector, 50 μL/well of diluted *R*-phycoerythrin-labeled Streptavidin conjugate (Invitrogen, Catalog No. S866) (1:250 dilution in PBS + 1% BSA+ 0.05% NaN_3_) was added and incubated for another 30 minutes. After washing, the beads were resuspended in 100 μL/well of PBS + 1% BSA + 0.05% NaN_3_ and analyzed using a Luminex 100 analyzer. The mean fluorescence intensity (MFI) from each well was determined by using Bio-Plex Manager Software, version 6.02 (Bio-Rad). The mean fluorescence intensity minus the signal intensity of the blank well was used in further analysis. Receiver operating characteristic (ROC) curves were used to illustrate assay performance[27].

#### Repeatability of the Luminex system

Intra- and inter-assay variability were determined for low- and medium-reactive control samples by testing 6 samples per run for 12 different times to produce 72 data points following the EP5-A2 (NCCLS) [28]. Low and medium reactive samples are constructed by dilution strong pooled positive sera using pooled negative human sera.

### TES-Ag western blot

TES-Ag proteins were electrophoretically separated using Criterion TGX (Bio-Rad, Cat. # 567–1092) at 10 ng/mm and then transferred to nitrocellulose membrane (Whatman Protran BA83, Cat. # 10 541 103, 0.2m pore size).The blots were cut into 2.5 mm strips and stored in PBS + 0.1% NaN_3_ at 4°C prior to use. Sera were tested and specific antibodies were detected as described previously [29, 30]. A serum was considered positive if reactivity occurred with any bands at 24, 28, 30, or 35 kDa [31, 32].

### Data analysis

Data were tabulated and analyzed using Microsoft Excel. Determination of the cut-off value and assay performance was obtained by using R statistical software version 3.0.1 (R Foundation for Statistical Computing, Vienna, Austria) and pROC package [33]. To combine the results of diagnostic tests, we used a method which finds optimal linear combination of multiple antigens[27]. In short, this procedure searches for the coefficients *a* and *b* in the equation below:

$$=a\;*\;{\text{MFI}}_{\text{Tc-CTL-}1}+b\;*\;{\text{MFI}}_{\text{Tc-TES-}26}$$

(MFI minus background is shortened to MFI in the equation for simplicity). In this formula, *a* and *b* are chosen to maximize the area under the ROC curve (AUC) and *y* is the combined value of the two MFI values. In order to avoid bias in the computation of the AUC, the AUC is estimated via cross-validation. Further details on the calculations can be found elsewhere [27].

## Results

### 2D gel mass spectrometry

**Fig 1:**
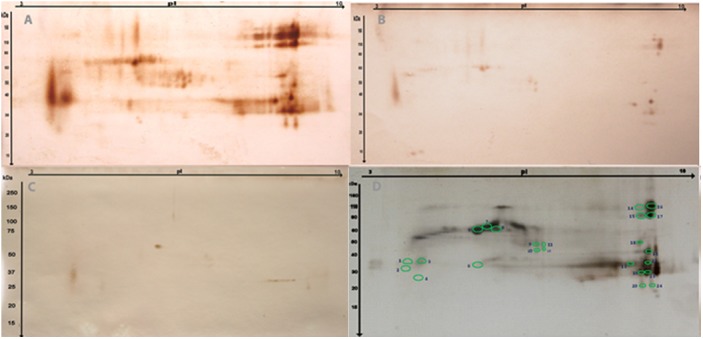
2-D gel electrophoresis, silver staining and western blotting of *Toxocara canis* Excretory Secretory Antigens (TES-Ag). The TES-Ag sample was separated and analyzed using 2D gel electrophoresis and western blotting. Three of the 2DE gels were transferred to nitrocellulose membranes and probed with a strong EIA positive *Toxocara* human sera pool (A), a negative human serum sample (B), and *Baylisascaris procyonis* positive serum (C).A reference gel was stained using silver stain (D). The circled spots in D represent proteins that were excised and subjected to mass spectrometry analysis.

From the 2D gel electrophoresis and mass spectrometry analysis, we identified 24 hits/spots that were reactive to *T*. *canis* positive serum, and lacked reactivity with both a *B*. *procyonis* positive baboon serum and a normal human serum (Fig 1A–1C). The corresponding protein spots were excised from the silver-stained gel and prepared for MS analysis (Fig 1D). An initial MASCOT search revealed 3 hits from spots 17, 22, and 23.The excretory/secretory mucin, MUC-3, from *T*. *canis*, was identified in spot 17, and excretory/secretory C-type lectin,Tc-CTL-1, from *T*. *canis*, at spots 22 and 23 (Table 1).When the MS data were used to search a *T*. *canis* Expressed Sequence Tag (EST) database, from 7 spots (2, 15, 17, 20, 22, 23, 24) we found nine mRNAs (Histone H4, Actin containing A3R Repeat, Actin, Tc-MUC-3, Fibrinogen beta and gamma chains, Glyceraldehyde 3-Phosphate Dehydrogenase, Elongation Factor, Tc-TES-26, Tc-CTL-1) with acceptable scores and percent sequence coverage. However, we identified only 3 proteins of interest (Tc-MUC-3, Tc-TES-26, and Tc-CTL-1) (Table 2). Tc-TES-26 from *T*. *canis* was identified as the protein in spot 20. The remaining 17 spots were determined to be human protein contaminants.

**Table 1:** Identified proteins from mass spectrometry analysis.

Spot	Accession	MW + (kDa)	Score	SC * (%)	*Toxocara canis-*related Protein
17	gi|5732922	27.9	84.6	8.9	Excretory/Secretory mucin MUC-3
22	gi|2773355	23.6	164.6	5.4	Excretory/Secretory C-type lectin CTL-1
23	gi|2773355	23.6	221.2	20.1	Excretory/Secretory C-type lectin CTL-1

Note

+ = Molecular Weight

* = Sequence Coverage

**Table 2:** Identified mRNA sequences from a *T*. *canis* expressed sequenced tag database.

Spot	Accession	MW + (kDa)	Score	SC * (%)	*Toxocara canis-*related Protein
2	AA873915.1	17.1	220.0	19.6	Histone H4
2	BQ274136.1	20.3	217.8	20.1	Actin containing A3R Repeat
2	HO243979.1	31.4	189.1	9.3	Actin
2	BQ458030.1	19.1	156.3	16	Actin
15	AA873915.1	17.1	220.2	19.6	Histon H4
15	BQ274136.1	20.1	155.9	16.2	Actin containing A3R Repeat
17	AA873915.1	17.1	220.2	19.6	Histon H4
17	AA728645.1	12.7	84.6	11.6	Tc-MUC-3 (mucin-3)
17	BQ274202.1	17.7	75.1	5.5	Fibrinogen beta and gamma chains
17	BM965454.1	19.0	741	8.2	Glyceraldehyde 3- Phosphate Dehydrogenase
17	BM088866.1	16.6	62.1	10.5	Elongation Factor
20	AA836708.3	19.2	52.6	4.0	Tc-TES-26
22	AI080928.1	12.7	164.6	20.2	Tc-CTL-1, c-type lectin
23	AI080928.1	12.7	221.2	38.6	Tc-CTL-1, c-type lectin
24	HO243979.1	31.4	386.1	22.1	Actin
24	BQ275419.1	22.3	372.2	31.7	Actin

Note

+ = Molecular Weight

* = Sequence Coverage

### Recombinant protein expression

**Fig 2:**
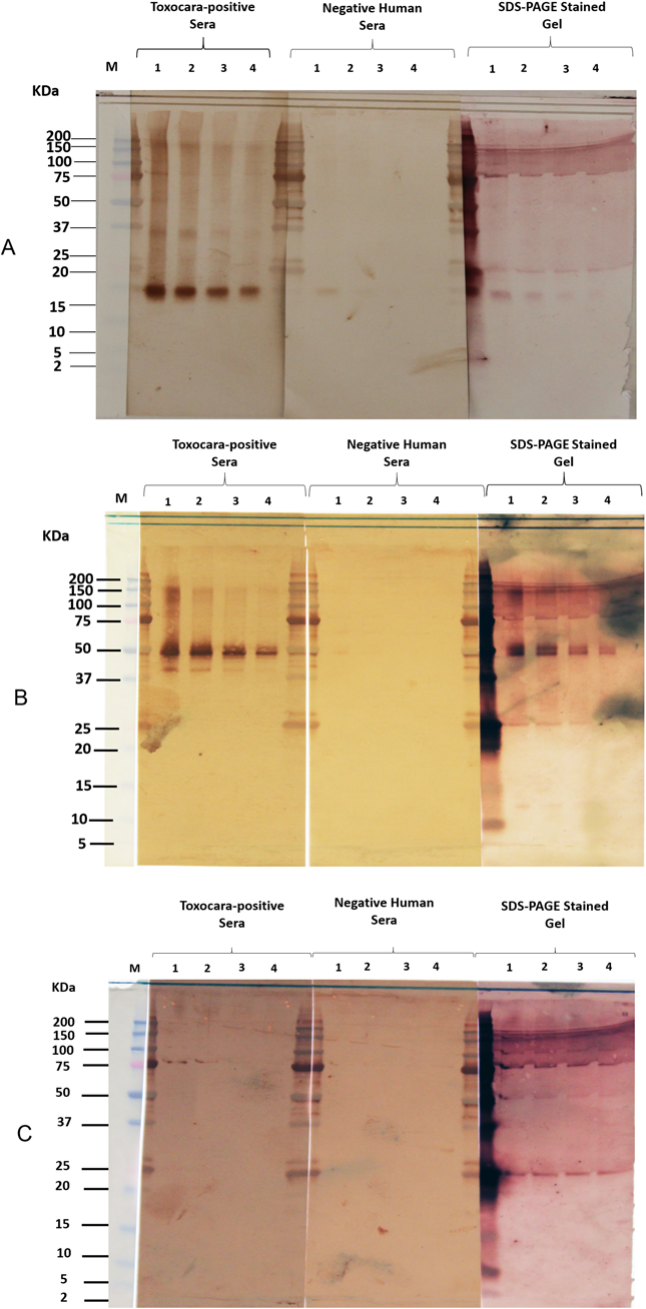
Purity and antigenicity of purified recombinant proteins. SDS-PAGE of the three recombinant proteins. A. Tc-CTL-1; B. Tc-TES-26; C. Tc-MUC-3. The recombinant protein samples at a concentration of: 1–6.25 ng/mm; 2–3.125 ng/mm; 3–1.6 ng/mm, and 4–0.8 ng/mm was treated with SDS and heated at 65°C for 15 minutes, separated and analyzed using SDS gel electrophoresis and western blotting. Two gels were transferred to nitrocellulose membranes and probed with a strong EIA positive *Toxocara* human sera pool, a negative human serum sample diluted 1:100 in PBS/Tween 0.3%/5% milk, and one gel was incubated with protein staining, Colloidal Gold Total Protein Stain (Bio-Rad, Cat. # 170–6527). M = Precision Plus Protein Dual Xtra Standards (Biorad, Cat. #161–0377)

We expressed Tc-CTL-1, Tc-TES-26, and Tc-MUC-3 in bacterial expression system. These three antigens were expressed as fusion proteins with GST tags to allow easy recombinant protein purification and also for improving the coupling possibility of the proteins to the magnetic beads. The purity and antigenic potential of the proteins could be seen in Fig 2. Among the three proteins, Tc-MUC-3 showed weaker reactivity when tested against the *T*. *canis* positive serum (Fig 2C).

### Luminex assay development

The three antigens, Tc-CTL-1, Tc-TES-26 and Tc-MUC-3, were selected for further evaluation in the Luminex assay based on the MS data. The Tc-MUC-3 antigen, after coupled to the magnetic beads, did not show differentiation between positive and negative sera although the protein performed well in the immunoblot format. No further analysis of Tc-MUC-3 was performed. Tc-CTL-1 and Tc-TES-26 were coupled to the MagPlex Magnetic Beads and Luminex based assays were developed. Each set of beads was tested using the defined serum batteries.

### Luminex assay performance

**Fig 3:**
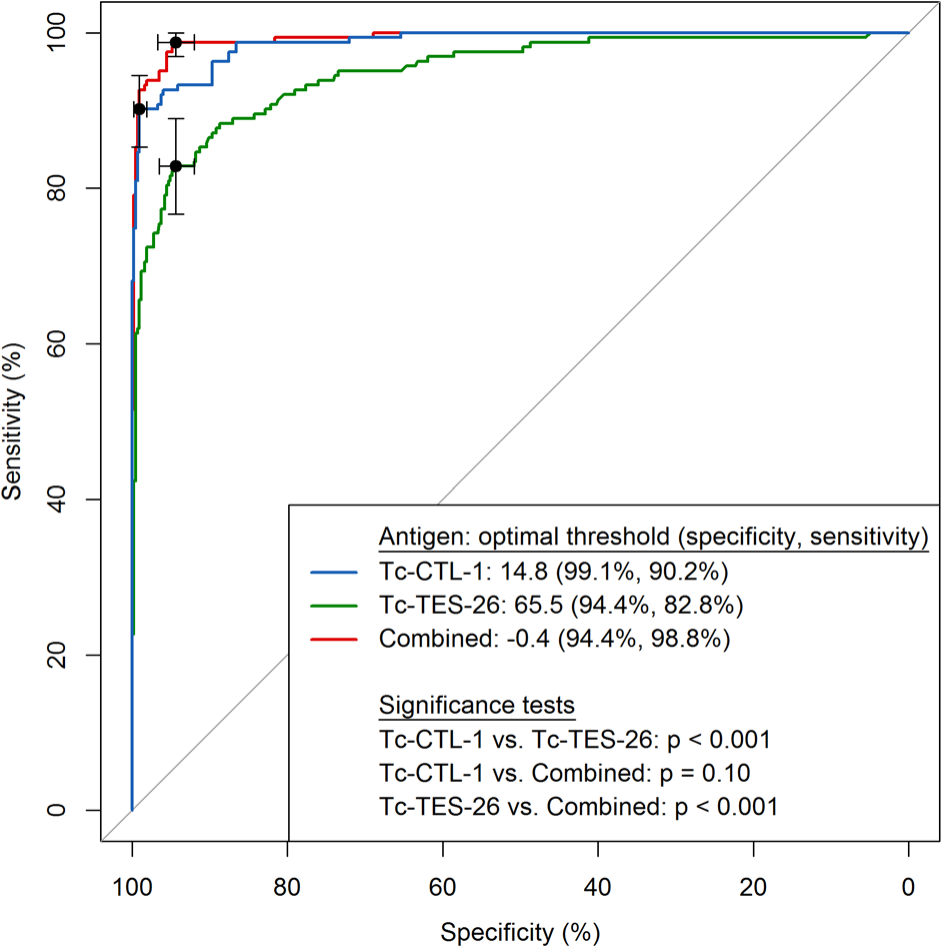
ROC Curves of Tc-CTL-1 and Tc-TES-26, and optimal linear combination for visceral larval migrans. ROC Curves of Tc-CTL-1, Tc-TES-26, and the optimal linear combination were constructed based on Luminex-derived mean fluorescence intensity antigen for visceral larval migrans from 288 negative U.S. serum samples + 134 heterologous parasitic infected serum samples and 204 visceral larval migrans positive serum samples; thresholds are calculated with the highest sum of sensitivity and specificity and are represented on the plot with black dots and corresponding 95% confidence intervals.

The Tc-CTL-1 Luminex assay performed significantly better than the Tc-TES-26 (p < 0.001) for diagnosis of visceral toxocariasis (Fig 3, Table 3). In comparison to the TES-Ag EIA, the Tc-CTL-1 Luminex assay also performed well for detecting visceral toxocariasis, however, only 54% of OLM cases were detected using the Tc-CTL-1 Luminex compared to 100% using the TES-Ag EIA (Table 3). We used the TES-Ag Western blot to better define the sera used from the OLM cases; only 70% of the OLM sera were reactive in the TES-Ag Western blot. When we restricted the OLM case definition to include only TES-Ag Western blot positive sera, the sensitivity and specificity increased 1% and 2% for Tc-CTL-1 and 3% and 3% for Tc-TES-26. To establish the specificity of the Luminex based assays, we used the sera from infections other than *Toxocara spp*. *The* Tc-TES-26 Luminex assay showed more cross-reactivity than the Tc-CTL-1 Luminex; only amebiasis and *E*. *nana* infections cross-react with Tc-CTL-1 (Table 4).

**Table 3:** Performance of Tc-CTL-1 and Tc-TES-26 Luminex based assays.

	Tc-CTL-1 (%) (95% CI)* (N)	Tc-TES-26 (%) (95% CI) (N)	Tc-CTL-1 plus Tc-TES-26	TES Ag-Western Blot (%)
Sensitivity				
**- visceral larval migrans**	90 (85–94) (204)	85 (79–89) (204)	99	100
**- ocular larval migrans**	54 (39–68) (50)	44 (30–59) (50)	64	70
**Specificity**	99 (97–100) (422)	91 (87–93) (422)	94	Not tested

Note

* = 95% Confidence Interval

**Table 4:** Specificity of the Toxocariasis Luminex assay.

Conditions	No. of Sera Tested	Tc-TES-26		Tc-CTL-1	
		No. of Positives	% Cross-reactivity	No. of Positives	% Cross-reactivity
US Negatives	288	20	7	2	0.7
Egyptian Negatives	14	2	14	0	0
Amebiasis	2	1	50	1	50
Ascariasis	6	2	33	0	0
Baylisascariasis	5	1	20	0	0
Clonorchiasis	3	0	0	0	0
Cryptosporidiasis	2	0	0	0	0
Cysticercosis	7	1	14	0	0
Dengue Fever	1	0	0	0	0
Echinococcosis	5	1	20	0	0
*E*. *nana*	2	2	100	1	50
Fasciolosis	2	0	0	0	0
Filariasis	3	0	0	0	0
Gnathostomiasis	2	0	0	0	0
Hepatitis	2	0	0	0	0
Hookworm	15	4	27	0	0
Hymenolopsis	8	0	0	0	0
Malaria	3	1	33	0	0
Paragonimiasis	4	1	25	0	0
Schistosomiasis	16	1	6	0	0
Strongyloidiasis	3	0	0	0	0
Taeniosis	5	0	0	0	0
Toxoplasmosis	5	0	0	0	0
Trichinellosis	12	2	17	0	0
Trichuriasis	6	2	33	0	0
Tuberculosis	1	0	0	0	0

Assay performance was improved when a combination of Tc-CTL-1 and Tc-TES-26 was used (Fig 3 and Table 3). The combination of the two antigens increased the sensitivity to 99% but lowered the specificity to 94% although this improvement was not statistically significant compared to Tc-CTL-1 (p = 0.10).

The coefficients of variation (CV) for intra-plate assays were 5% for Tc-CTL-1 and 4% for Tc-TES-26. For inter-plate variation, the CV for the low positive control was quite large at 23% for Tc-CTL-1 and 31% for Tc-TES-26, probably because of the low value of the observations; but for medium calibrator, the CV was 7% for Tc-CTL-1 and 12% for Tc-TES-26.

## Discussion

The study confirmed previous studies on immunodominant TES antigens [1, 13, 16, 19, 20, 23, 24]. Briefly, we identified three major antigenic proteins from the TES-Ag, and based on these findings, expressed those antigens for further analysis as diagnostic reagents. One antigen, Tc-MUC-3, after coupled to Luminex beads, did not produce a functional assay. We have no explanation for this, as we tried several methods of coupling including titration of antigens, different buffers, and pH conditions. It is possible that the low reactivity of this protein to positive sample causes the failure for coupling the protein to the beads.

The performances of Tc-CTL-1 and Tc-TES-26 based on total IgG responses are comparable to the reported performances of the same antigens. Yamasaki et al. (2000) [16], based on 11 subjects with toxocariasis, reported a sensitivity of 100% for Tc-CTL-1 and a specificity of 98% (3 out of 142 cross-reactors sera). From the study of Mohamad et al. (2009) [15], and Norhaida et al. (2008) [34], the sensitivity and the specificity of Tc-CTL-1 is 92–93% and 94–90%, respectively. The Tc-CTL-1 in our study has a sensitivity of 90% and a specificity of 99%. The differences of the performances of the assays due to a possibility that Yamasaki study used small number of positive sera and also soil-transmitted helminths were not prevalent in Japan. A Low prevalence of soil-transmitted helminths might contribute to less background or reactivity to the assay and will lower the cut-off points, thereby improving the sensitivity. In Norhaida study, the group used IgG_4_, instead of total IgG [34]. For Tc-TES-26, Mohamad et al. (2009) [15] reported the assay has a sensitivity of 80% and specificity of 96% (similar to Tc-TES-26 Luminex performance), but this performance was based on IgG_4_ responses. Our assay was not based on specific IgG_4_ detection and it is possible that we could improve the sensitivity and specificity of the assay if we were to use the IgG_4_ specific responses [15, 34].

Recombinant-based assays to determine antibody responses usually have lower sensitivity than those based on native/crude antigens because reactivity with multiple antigens can occur when crude mixtures are used. As expected, the assays developed here using Tc-CTL-1 and Tc-TES-26, are less sensitive than the TES-Ag EIA or TES-Ag Western Blot, which utilize a crude parasite antigen. However, the combination of the two antigens resulted in an improved sensitivity of 99% for VLM detection that equaled the sensitivity of the TES-Ag Western Blot. Not unexpectedly, when sensitivity was maximized, the assay specificity decreased to 94% when the combination of two antigens was used. This specificity is not optimum, but it is still much better than the reported specificities of TES-Ag EIAs which are approximately 85% [4]. Although the Luminex based assays all represented improvements in detecting VLM, all were less sensitive than the TES-Ag EIA or even the TES-Ag Western blot method for detecting OLM cases. While additional antigens might improve the sensitivity for detection of OLM, we wonder how many individual antigens might be needed since the TES-Ag western blot, which is a complex mixture of multiple antigens failed to detect 30% of OLM subjects. Compared to the reference TES-Ag EIA, the use of the Luminex platform has advantages: more samples could be tested concurrently and downstream, a multiplex assay could be developed to distinguish human larval migrans syndromes caused by other helminths such as *B*. *procyonis*, another important cause of larval migrans in the U.S., as well as other larval helminths species [2, 35].

Although the Luminex-based assay offers advantages against the EIA (in term of performance and multiplexing capability), the capital cost is much higher than for an EIA [36]. If the system is only used for detecting responses against single antigen, the Luminex-based assay is not cost effective and does not offer advantage on time-saving. For each additional antigen, the time and cost savings for running the Luminex-based assay are much better than running several EIA. The capital cost for running Luminex-based assay even could be reduced further by using a more robust, field-friendly system of MagPix that has similar performance to the Luminex platform. The only disadvantage of MagPix is that the capability for multiplexing is only up to 50 antigens (not 100 antigens as for the Luminex system). In the term of skill needed, Luminex-based assay is similar to the requirement of the skill for running an EIA.

## Conclusions

Recombinant antigen based assays in the Luminex platform for visceral toxocariasis perform similarly to the existing TES-Ag Western Blot and better than the TES-Ag EIA method. The utilization of the Luminex assay significantly diminishes the need for native parasite materials which can be expensive and cause data variability.
